# Tau Protein Biosensors in the Diagnosis of Neurodegenerative Diseases

**DOI:** 10.34172/apb.2023.061

**Published:** 2022-11-04

**Authors:** Jafar Sadeghzadeh, Parviz Shahabi, Mehdi Farhoudi, Abbas Ebrahimi-Kalan, Ahmad Mobed, Kourosh Shahpasand

**Affiliations:** ^1^Department of Neurosciences and Cognition, Faculty of Advanced Medical Sciences, Tabriz University of Medical Sciences,Tabriz, Iran.; ^2^Department of Physiology, School of Medicine, Tabriz University of Medical Sciences, Tabriz, Iran.; ^3^Neuroscience Research Center, Tabriz University of Medical Sciences, Tabriz, Iran.; ^4^Physical Medicine and Rehabilitation Research Center, Tabriz University of Medical Sciences, Tabriz, Iran.; ^5^Department of Stem Cells and Developmental Biology, Cell Science Research Center, Royan Institute for Stem Cell Biology and Technology (RI-SCBT), Tehran, Iran.

**Keywords:** Neurodegenerative disease, Tau protein, Biosensors, Clinical diagnosis

## Abstract

Tau protein plays a crucial role in diagnosing neurodegenerative diseases. However, performing an assay to detect tau protein on a nanoscale is a great challenge for early diagnosis of diseases. Enzyme-linked immunosorbent assay (ELISA), western-blotting, and molecular-based methods, e.g., PCR and real-time PCR, are the most widely used methods for detecting tau protein. These methods are subject to certain limitations: the need for advanced equipment, low sensitivity, and specificity, to name a few. With the above said, it is necessary to discover advanced and novel methods for monitoring tau protein. Counted among remarkable approaches adopted by researchers, biosensors can largely eliminate the difficulties and limitations associated with conventional methods. The main objective of the present study is to review the latest biosensors developed to detect the tau protein. Furthermore, the problems and limitations of conventional diagnosis methods were discussed in detail.

## Introduction

 Tau protein was first identified by Weingarten et al in 1975.^1^ Known as a heat-stable protein essential for microtubule assembly,^2^ Tau belongs to the family of microtubule-associated proteins (MAPs). Stabilization of microtubules, which are the central part of cytoskeleton scaffolds in supporting cellular trafficking and cells, plays a key role in tau protein detection.^2^ Normal tau is usually found in axons. In the case of diseases affecting tau function and structure, tau protein can be translocated in the dendrites and cell body.^3^ Abnormal deposition of the modified tau proteins in neurons is a common feature of several neurodegenerative diseases known as “tauopathies”.^4^ Since tau is actively secreted by neurons, in both post-translational modifications (PTMs) and specific unmodified states, ultra-sensitive identification of tau as the predictor of Alzheimer disease (AD) symptoms can be a challenging task.^5,6^ In addition, the total level of tau in CSF correlates with neurodegeneration in AD, which makes it a golden biomarker of neurodegenerative diseases such as AD.^7^ Tau detection is usually carried out using immunoassays such as enzyme-linked immunosorbent Assay (ELISA). However, this technique suffers some inherent disadvantages such as time-consuming operation, but antigen information, and temporary readouts.^8,9^ Therefore, given the significance of tau protein and limitations of the corresponding identification methods, a number of researchers have emphasized the development of advanced and highly sensitive methods. Nanobiotechnology is one of the most significant fields in the development of new diagnostic methods that has received considerable attention from scientists in recent years.^10,11^ Biosensors are one of the main branches of nanobiotechnology whose development can overcome the difficulties and limitations associated with the routine methods.^11^

 The present study is structured as follows: the first part is dedicated to the study of tau protein and tauopathy; the second part discusses the role of routine methods in the diagnosis of tau protein and tauopathy; and the third part introduces the developed biosensors used for detecting tau protein and tauopathy.

## Tau protein pathophysiology

**Figure 1:**
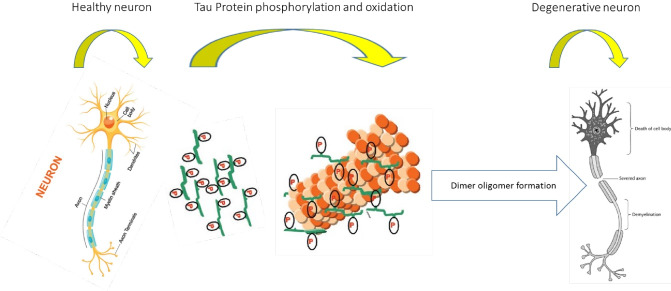
Schematic illustration of the central role of tau oligomers in tauopathies

 For the first time, Gloria Lee et al elaborated on tau structure.^12,13^ They pointed out that the sequence of tau was not similar to its C-terminus that had a certain repeated sequence, which was assumed to be a tubulin binding site.^7,14^ Tau protein can be prearranged in C-terminal, N-terminal, and repeat domains. Tau is neutrally charged in the basic repeat region, N-terminal acidic region, and neutral C-terminal region.^7,14^ Normally, the brain comprises intact neurons that facilitate communication between nerve impulses and cooperation with the adjacent neurons, thus establishing a neural network. The structure of a neuron is preserved by cytoskeletal proteins.^7,14^ The axonal micro-tubule (MT) is stabilized by tau protein, which also preserves the shape of axon.^15^ In AD, tau proteins are exposed to several PTMs that reduce the affinity of tau with MT and instead, they accumulate to form aggregates.^15,16^ On the contrary, depolymerization of microtubules and loss of axonal correlation lead to degeneration, which is a feature of shrunken brains.^16^ Figure 1 presents a schematic illustration of the tauopathy presented.^15^

 In this figure, while tau in a healthy brain regulates microtubule stabilization, tau hyperphosphorylation causes microtubule affinity loss in tauopathies. Soluble tau aggregates in pathological soluble tau oligomers, thus forming pathological insoluble neuro-fibrillary tangles.^17,18^ Tau oligomers are secreted into the extracellular compartment, facilitating the transmission of tau pathology into neighboring neurons. Inflammatory inducements, such as Aβ, stimulate the microglial production of pro-inflammatory mediators such as IL-1β, leading to the up-regulation of kinases involved in tau phosphorylation and exacerbation of the pathology.^17,18^ However, inflammation can have beneficial effects on tau pathology by inducing microglial phagocytosis of extracellular tau species.^17,18^

###  Conventional methods for tau identification 

 Immunoassay is based on specific antigen-antibody binding including western blot, ELISA, and immuno-magnetic reduction.^19^ ELISA is one of the most important methods of detecting tau protein. Although it is used for tau protein detection via a simple operation, it is still time-consuming and requires advanced tools.^20^ Capillary electrophoresis (CE) based enzymatic assays were employed to identify tau protein in human serum. The CE method highlights the role of tau in developing the operative medicines for neurodegenerative disease treatments.^21^ A single-molecule enzyme-linked immunosorbent assay was also developed to detect serum proteins at subfemtomolar concentrations. The created system is characterized by sufficient sensitivity and specificity to routine methods.^22^ Being a powerful method with few sample treatment requirements, high sensitivity, and immediate identifying capability, mass spectrometry was applied for determining tau protein as a critical neurodegenerative disease biomarker.^23^ In general, routine methods for diagnosing tau protein have certain advantages and disadvantages, as illustrated in Table 1.

**Table 1:** Conventional and widely used methods for detecting tau protein

**Techniques**	**Sample type**	**Advantages and potentials**	**Disadvantages and limitations**	**Ref**
ELISA	Plasma, Tissue	Fast, proper, very sensitive, and specific. Reagents are relatively inexpensive and have a long shelf life. This device is relatively low-cost and extensively used.	Cross reactivity, Challenging for detection of sum biomarker such as microRNAs, and not applicable to nanoscale samples.	^24,25^
Mass spectrometry	Tissue	Analysis of thousands of proteins, automation, multidimensions, fast, high sensitivity, small sample volume, multidimensional, and extremely multiplex. Detectable protein isoforms.	Time-consuming, sensitive to interfering compounds, restricted mass range, complex sample preparation, poor analytical sensitivity compared to immunoassays, and low-throughput need for relatively expensive tools.	^26,27^
Western blotting	Mouse brain	Separation of proteins by molecular weight.	Work-intensive, medium-throughput; background resulting from cross reactivity of antibodies; gel preparation being time consuming; and large amount of protein required for detection.	^28,29^
PCR and real-time PCR	CSF	Specific, sensitive, and fast. Precision in discovery of small amounts of target nucleic acid.	No distinction between dead or alive cells, smear bond, and primer dimer band.	^30-32^

## Biosensor technology

 Biosensors are analytical devices equipped with biological elements, such as antibodies and nucleic acids, and electronic components in order to generate quantifiable signals.^33,34^ Electronic components detect, record, and transmit information about the presence of several physiological and chemical changes or biological substances in the environment.^33,34^ Biosensors come in different shapes and sizes and can be detected and measured under such conditions as low concentrations of biomarkers, specific pathogens, toxic chemicals, and pH levels. In general, biosensors include transducers, analytes, bioreceptors, electronic devices, and monitors.^33,34^

**Figure 2:**
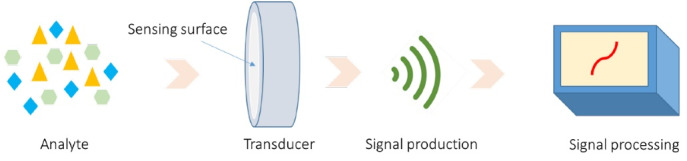
Schematic illustration of biosensor technology

 Bio-recognition is the process of signal production during the interaction between the bio-receptor and analyte. Transducer is a device that transforms energy from one form into another.^35,36^ As shown in Figure 2, the transducer is the main element in a biosensor that converts the bio-recognition occurrence into a measurable electrical signal and this happens in the presence of a chemical or biological target.^35,36^ The process of energy conversion is identified as signalization. Moreover, transducers generate either electrical or optical signals comparable to the number of analyte–bioreceptor interactions.^35,36^ Given the working principle, transducers are generally classified as electrochemical, optical, and mechanical ones. The electrical signals acquired from the transducer are amplified and converted into digital forms.^35,36^ The processed signals are quantified by the display unit. Display part is composed of a user interpretation system, such as a computer or a printer, which creates the output so that the corresponding response can be comprehensible and readable by the user.^36,37^

**Figure 3:**
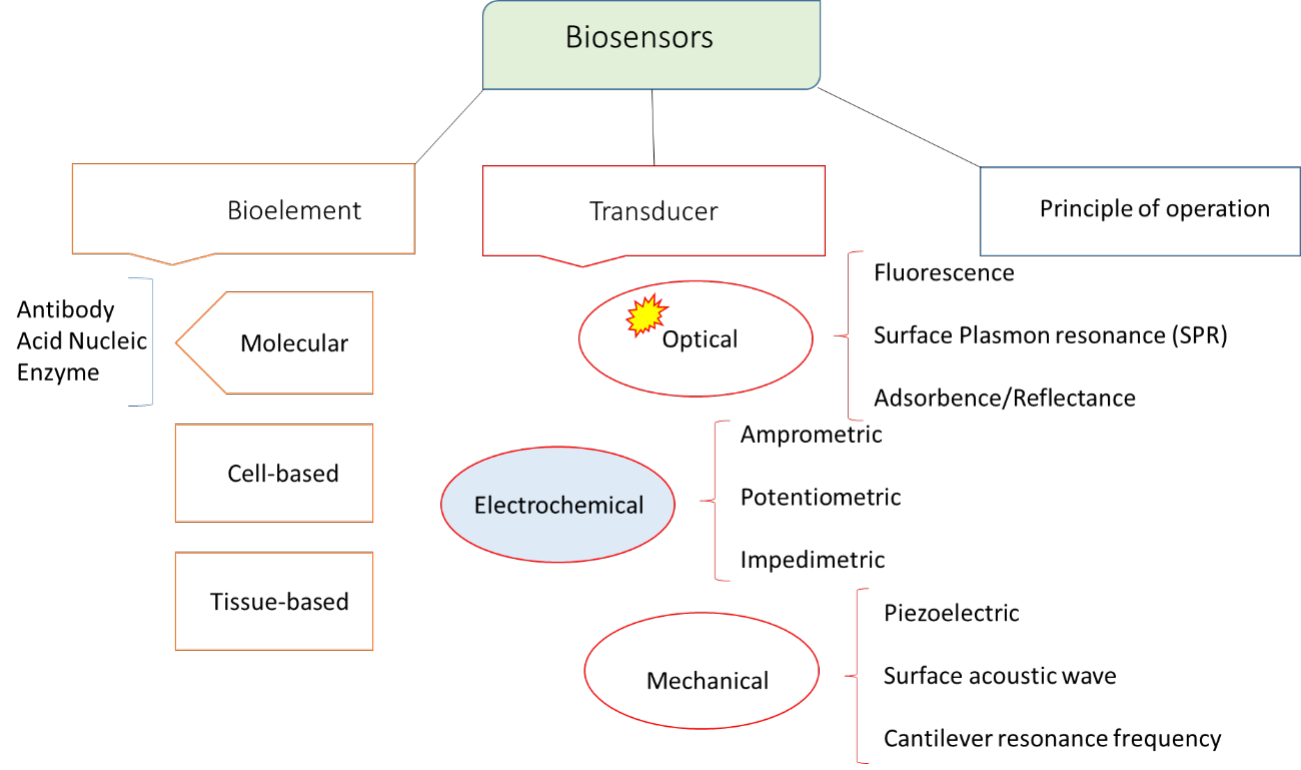
Schematic illustration of biosensor classification

**Figure 4:**
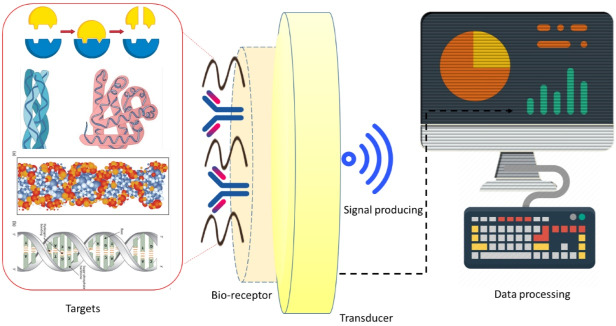
Optical biosensors measure changes in a particular characteristic of light, coupled to changes linked to binding at the sensor surface by making use of the electromagnetic evanescent wave formed at the gold interface of the sensor chip surface

**Figure 5:**
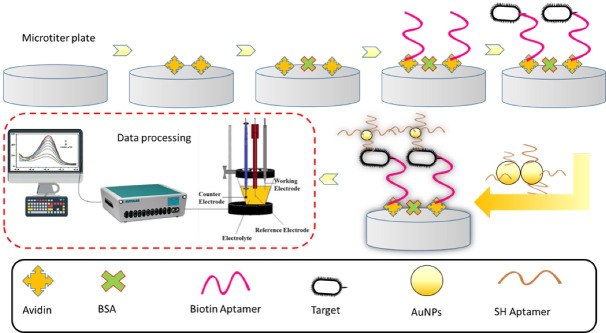
Electrochemical biosensor, in this type of sensor, an electrode is a key component that is employed as a solid support for immobilization of biomolecules and electron movement. Derived from Dridi et al and Chen et al^39,40^

 As observed in Figure 3, based on the type of transducer, biosensors are classified as optical, electrochemical, and mechanical ones. Amongst them, optical and electrochemical biosensors are extensively used for determining different biomarkers.^34^ In sum, biosensors are characterized by high sensitivity, wide dynamic range, small and simple structure, applicability to remote sensing, immunity to electromagnetic interference, capability to monitor a wide range of biomolecules, and consistent operation.^37,38^ Figures 4 and 5 include schematic images of electrochemical and optical biosensors.

###  Developed biosensors for tau protein identification 

####  Electrochemical biosensor 

**Figure 6:**
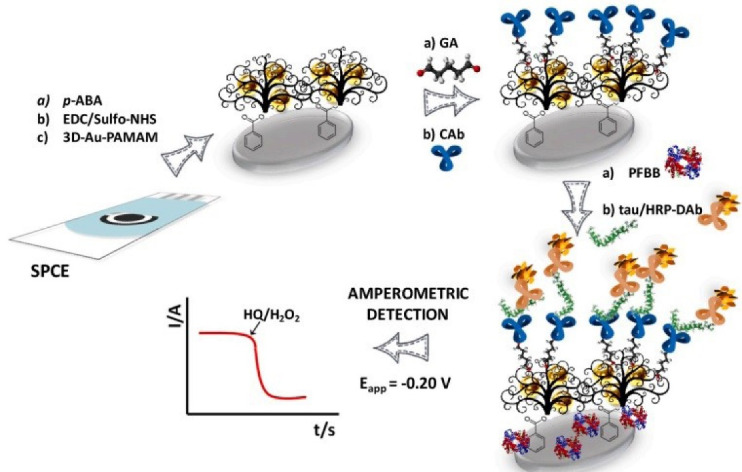
Fabrication and amperometric transduction involved in the development of a HRP-DAb-tau-CAb-3D-Au-PAMAM-p-ABA-SPCE immunosensor for tau protein determination. Reprinted with permission from Razzino et al^41^

 An electrochemical immunosensor platform based on gold nanoparticles and polyamidoamine (PAMAM) dendrimer, screen-printed carbon electrodes were developed for accurate and specific identification of tau protein in biological samples as well as brain tissues suffering neurodegenerative diseases. Figure 6 depicts the created biosystem featuring high sensitivity, simple and cost-effective structure, and wide linearity range.^41^

 A protein-based electrochemical biosensor was developed for selective detection of tau in PBS solution. The created biosensor is able to distinguish the normal tau protein from the abnormal one; therefore, it can be used to deal with the problem of neurodegenerative diseases.^42^ A simple and low-cost biosensor was established for highly selective detection of tau protein in AD. The generated immunosensor exhibited acceptable sensitivity and wide range of linearity.^43^

 The Aptamer-based biosensor is settled for precise and specific recognition of tau protein. This system can provide an alternative method for tau protein phosphorylation and aggregation in the PBS solution.^44^

 To diagnose tau protein, an electrochemical biosensor was employed as a reliable tool. The advanced bio-system exhibited good sensitivity and fast operation, hence being applicable to AD screening in care settings.^45^ A multi-amplified electrochemical biosensor was invented to identify tau protein in the dementia patient. The proved biosensor displayed adequate reproducibility, specificity, and stability. The technique was effectively applied to the biological samples such as serum of the normal people, mild cognitive impairment patients, and dementia patients.^46^ An electrochemical immunosensor was assembled to detect tau protein in biological fluids as a neurodegenerative disease biomarker. This engineered electrochemical sensor exhibited good sensitivity and selectivity followed by a reliable result for clinical approach.^47^ An innovative label-free electrochemical aptasensor was developed for determining tau protein in biological samples. The proposed aptasensor can also be applied to the clinical diagnosis of AD at early stages with high selectivity.^48^

 An electrochemical immunosensor was organized to detect tau protein in the biological samples. According to the reports, the designed system was characterized by a wide linearity range and high sensitivity with fast operation. The planned biosensor may be used as a substitute test for screening AD and neurodegenerative diseases.^49^ To ensure a rapid, simple, ultra-sensitive, and specific measurement of tau protein in human serum, a novel electrochemical aptamer-antibody sandwich assay was appropriately assembled. Despite the simple and low-cost structure, the designed system was characterized by proper analytical features.^50^

####  Fluorescence and SPR-based biosensors

**Figure 7:**
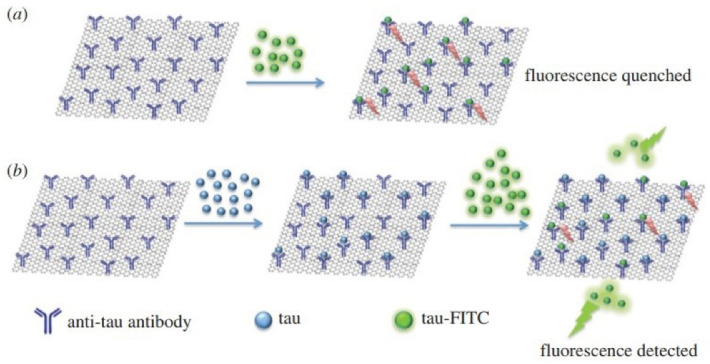
Schematic of GO-based fluorescence quenching for the detection of tau protein. (a) The fluorescence signal of tau-FITC will be quenched when there is no analyte tau in solution. (b) When analyte tau protein molecules are present and adsorb on antibody-conjugated GO, fewer tau-FITC are adsorbed and quenched. Thus, the fluorescence signal from free tau-FITC in buffer correlates with analyte tau concentration (see Huang et al^51^; the Creative Commons Attribution License CC BY 4.0).

 An innovative immunosensor based on the controlled ﬂuorescence quenching was set up for specific detection of tau protein. In this study, graphene oxide was employed as a cost-effective nanomaterial for selective diagnosis of tau protein in neurodegeneration syndromes.^51^ A schematic illustration of the created system is represented in Figure 7.

 Surface plasmon resonance (SPR) immunosensor was designed for accurate and specific recognition of tau protein in AD. The obtained results exhibited high stability and signal repeatability in CSF samples with acceptable LOD and linearity.^52^ Detection of atypical tau protein is essential for screening for degenerative diseases. To this end, a localized surface plasmon resonance-based immune-chip-based biosensor should be appropriately developed. Suitable sensitivity and selectivity with acceptable linearity was reported from the settled LSPR biosensor.^53^

**Figure 8:**
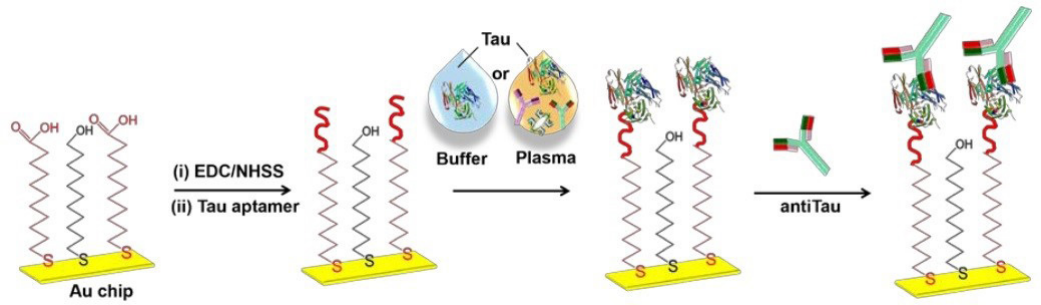
Schematic overview of strategy for Tau protein detection. Mixed monolayers of MUA and MUD were first immobilized on a SPR chip followed by the covalent attachment of DNA aptamer probes. Specific adsorption of Tau proteins was monitored in both buffer and plasma followed by the subsequent binding of antiTau. Reprinted with permission from Kim et al^54^

 The SPR-based biosensor was also developed for ultra-sensitive detection of tau protein as the main biomarker in neurodegenerative diseases. The designed system, as presented in Figure 8, enjoys greater selectivity and specificity than other routine tests such as ELISA.^54^

 Blood-based immunoassay using surface plasmon resonance fiber sensors was advanced for tau proteins as point-of-care testing. The adjusted system facilitates early diagnosis of AD with high selectivity and sensitivity in a minimum span of time.^55^ Fluorescence-based immunoassay using dopamine-functionalized CuInS_2_/ZnS quantum dots was applied for detection of tau protein. Based on the obtained results, the engineering platform, can be used as the best alternative to conventional method as well as ELISA.^56^

 The LSPR-based biosensor functionalized by GNPs is applied for accurate and selective detection of tau protein. The presented system can be used as a low-cost and simple method for monitoring AD and other neurodegenerative disease biomarkers.^57^

 A novel SPR-based biosensor was planned for rapid and sensitive determination of tau and amyloid β (tau-Aβ) complex in CSF samples. The engineered bio-device organized as a sandwich assay incorporating functionalized AuNPs allows for the detection of the tau-Aβ complex with good LOD and wide linearity range.^58^ An ultra-sensitive aptamer-assisted amplification method was established for the identification of tau protein in AD. The engineered bio-device can be employed for selective detection of abnormal tau protein from beta-amyloid protein.^59^

####  Colorimetric biosensors

**Figure 9:**
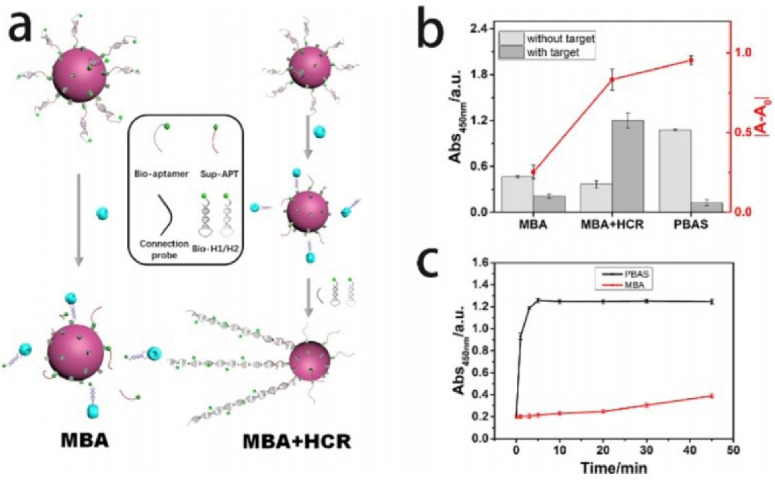
(a) Schematic illustration of MBA-based structures for the detection of tau. (b) Peak values of TMB UV−vis absorption spectra detecting 20 μg/mL tau using different nanostructures. (c) Time required for the capture of the PBAS and MBA by strep-MB, with permission from ref (see Duan et al^60^)

 Aptamer-based platform is designed for rapid and selective determination of tau protein in the case of neurodegenerative diseases. The developed method works on the basis of colorimetric analysis and has been designed to be simple and inexpensive in diagnosing tau protein in related diseases^60^ (Figure 9).

####  Other biosensors

 Beta-amyloid and total tau were measured by the established novel multiplexed biosensor in CSF. The created bio-device had a simple and inexpensive structure and easy operation for nano-scale samples.^61^ The functions of myelin and tau proteins as critical biomarkers of AD and multiple sclerosis were measured in CSF fluid properly. The used nanoimmunosensor exhibited higher sensitivity and specificity than the ELISA method.^62^ Magnetic nanoparticles and liquid-based immunosensor were applied in conjunction for tau protein determination. The designed platform had more than 90% sensitivity and specificity to all the human blood samples.^63^ For real-time detection of tau protein, a Quartz Crystal Balance (QCM) immunosensor was settled successfully. The planned system displayed promising advantages for studying tubulin polymerization in AD diagnosis and monitoring.^64^

Table 2 includes the following highlights. (A) In most studies, gold nanoparticles have been used because of their valuable properties such as high conductivity. (B) Although gold nanoparticles can increase the sensitivity and specificity of biosensors, they can be costly; therefore, the use of inexpensive nanomaterials such as carbon-based nanomaterials should receive greater attention. (C) Despite the growing development of biosensors for different types of biomarkers, few biosensors have been developed in connection with the tau protein; therefore, their expansion via different techniques can be valuable. (D) Paper-based biosensors are simple and inexpensive tools that have been developed in conjunction with some biomarkers. It appears that the expansion of paper-based biosensors could be one of the most attractive objectives for researchers in future research.

**Table 2:** Developed biosensors for tau protein detection

**Platform**	**Technique**	**Nanomaterial**	**Linear range**	**LOD**	**Ref**
Sandwich immunoassay	Amperometry	3D-Au-PAMAM	6-5000 pg mL 1 (~0.11-91 pM)	1.7 pg mL 1	^41^
Immunosensor	Fluorescence quenching	GO	0 to 20 ng mL^-1^	0.14 pmol mL^−1^	^51^
Immunosensor	Electrochemistry	Au sputtered silicon wafers	0.2 to 1.0 µM	_	^42^
Immunosensor	SERS	MNP	25 fM to 500 nM	25 fM	^43^
Aptasensor	Optical spectroscopy	GNPs	0.005–1 μM	6.7 nM	^44^
Immunosensor	SPR	MWCNTs/GNPs	125.0 nM 31.2 nM	2 nM	^52^
Aptasensor	Colorimetric	Strep-MBs	0.2−2000 ng mL^−1^	153 pg mL^−1^	^60^
Immunosensor	Impedance	PVDF	(10-14 M to 10-7 M	0.03 pM	^45^
Immunosensor	LSPR	GNPs/Silica	195 pg/mL	10 pg/mL	^53^
Immunosensor	Optical	AAO	400 pg/mL	15.6 pg/mL	^61^
Immunosensors	Electrochemical	rGO /MWCNTs/ AuNPs	0.5–80 fM	0.46 fM	^65^
Immunosensor	Impedance spectroscopy	Gold	100 μg/mL	_	^47^
Immunosensor	SPR	Gold chip	10 fM	10 pM	^54^
Immunosensor	EIS, DPV	GO	-	0.15 nM	^62^
Immunosensor	SPR	Gold	0 to 4360 pg mL^−1^	1.6 pg mL^−1^	^55^
Immunosensor	SPR	CuInS2/ZnS quantum dots	10 pM to 200 nM	9.3 pM	^56^
Immunosensor	Fluorescent Labeling	MNP	100 ng/mL	100 ng/mL	^63^
Aptasensor	Electrochemical	CGTGNPs	1.0 pM to 100 pM	0.70 pM	^48^
Aptasensor	TIRFM -EMCCD	IONPs	0-1000 fM	165 fg/mL	^59^
Immunosensor	QCM	MPN	30-125 nM	34 ng L^−1^	^64^
Immunosensor	Electrochemical	Gold	1000 pg/mL to 100,000 pg/mL	-	^49^
Immunosensor	LSPR	GNPs	1. 10^1^ fM to 1. 10^8^ fM	23.6 fM	^57^
Immunosensor	SPR	GNPs	up to ∼5 pM	1 pM	^58^
Aptamar-antibody	Electrochemical	AuNPs	0.5 pM to 100 pM	0.42 pM	^50^

(GO): graphene oxide, (SERS): surface-enhanced Raman scattering, (MNP): magnetic nanoparticle, (MWCNTs): Multi Walled Carbon Nanotubes, (GNPs): Gold nanoparticles, (Strep-MBs): Streptavidin-coated magnetic beads, (PVDF): Polyvinylidene difluoride, (LSPR): localized surface Plasmon resonance, (AAO): anodic aluminum oxide, (EIS): electrochemical impedance spectroscopy, (CGTGNPs): carboxyl graphene/thionin/gold nanoparticle, (TIRFM-EMCCD): total internal reflection fluorescence microscopy electron-multiplying charge-coupled device. (IONPs): Iron oxide nanoparticles.

 Finally, although all diagnostic methods have inherent disadvantages and advantages, biosensors seem to be more valuable methods than conventional methods. Furthermore, the improvement of expanded biosensors can promise advanced and flawless method.

## Conclusion

 According to our findings, tau protein plays an essential role in the detection of neurodegenerative diseases. Therefore, a specific and rapid diagnosis is quite instrumental in controlling and even treating the disease. The use of biosensor technology for the detection of tau has not been fully implemented or discovered. Most of the developed biosensors are still functional in the realm of research and not yet applicable to real samples. Therefore, a significant deal of effort is still required to reach more reliable and improved devices to ensure ultra-sensitive detection of tau protein in real and biological samples. Furthermore, the existing interest and the expected advancements in nanotechnology research will possibly allow the development of promising bio-devices. So far, a biosensor that can both replace the routine approaches to tau protein detection and circumvent the limitations of these approaches remains inaccessible; hence, further research and attention is required to develop such a particular type of biosensor.

## Acknowledgments

 The authors would like to thank the Aging Research Institute and the Physical Medicine Rehabilitation Research Center of Tabriz University of Medical Sciences. This work is part of PhD thesis of Jafar Sadeghzadeh.

## Competing Interests

 The authors declare that they have no known competing financial interests or personal relationships that could appear to influence the work reported in this paper.

## Ethical Approval

 Not applicable.
